# Is computed tomography assessment of residual arterial pedicle length following colorectal cancer surgery a useful marker of surgical quality?

**DOI:** 10.1007/s10151-025-03130-6

**Published:** 2025-04-12

**Authors:** K. Naidu, P. Chapuis, J. Yang, S. Koneru, C. Chan, M. Rickard, K.-S. Ng

**Affiliations:** 1 Colorectal Surgery Unit, Concord Hospital, Concord, NSW 2139 Australia; 2 Concord Institute of Academic Surgery, Concord Repatriation General Hospital, Building 20, Level 1, Hospital Road, Concord, NSW 2139 Australia; 3 Concord Clinical School, Clinical Sciences Building, Concord Hospital, University of Sydney, Concord, NSW 2139 Australia; 4 Department of Radiology, Concord Hospital, Concord, NSW 2139 Australia; 5 Department of Anatomical Pathology, Concord Hospital, Concord, NSW 2139 Australia

**Keywords:** CME, CVL, Residual arterial pedicle length, Tissue morphometry

## Abstract

**Background:**

*In vivo* residual arterial pedicle length (RAPL) has been proposed as a quality indicator for central vascular ligation (CVL [i.e., RAPL ≤ 10 mm]) in colorectal cancer (CRC) surgery. However, its survival association in non-routine CVL practice requires clarification. This study aimed to assess the feasibility and reproducibility of measuring RAPL alongside its oncological associations in non-routine CVL surgery.

**Methods:**

A prospective cohort study at Concord Hospital was conducted on anterior resection (AR) or right hemicolectomy (RH) patients with stage I to III CRC (1995–2019). Using surveillance computed tomography (CT), RAPL of the inferior mesenteric artery (IMA) or ileo-colic artery (ICA) pedicle was measured independently by two observers. The intra-class correlation coefficient assessed the reproducibility of the measurements. Kaplan-Meier and univariate Cox regression analyses estimated overall survival (OS) and disease-free survival (DFS), while univariate and multivariate linear regression models tested correlations between RAPL and clinicopathological features.

**Results:**

A total of 1425 patients underwent a CRC operation. Post-operative CTs were reviewed in 424 patients, with 422 (mean age 69.0 years [SD 12.3]; 54.0% males) RAPLs measured. The majority studied underwent an AR (59.2%). Excellent inter-rater reliability was noted in AR (ICC = 0.97; *P* < 0.001) and RH (ICC = 0.89; *P* < 0.001) patients. No association was observed between RAPL and OS or DFS in either group. Also, RAPL lacked association with nodal harvest in either AR (*P* = 0.54) or RH (*P* = 0.16) patients.

**Conclusion:**

The value of RAPL as a quality marker of CRC surgery in non-routine CVL practice has not been confirmed. Furthermore, its lack of association with nodal harvest emphasizes the importance and the need for comprehensive pathology examination of the specimen following resection of CRC.

## Introduction

Central tenets of ‘good quality’ CRC surgery include operating in the correct anatomical plane, complete primary tumour removal with clear margins, and performing an adequate lymphadenectomy. Most early studies appraising surgical quality have centred on the correct plane of excision (total mesorectal excision [TME] for rectal cancer or complete mesocolic excision [CME] for colon cancer) and avoidance of tumour transection [1–5]. However, the extent of lymphadenectomy (EoL) has arguably received less attention. This may be due to challenges in its objective measurement, which is so far limited to the rudimentary assessment of lymph node (LN) harvest in the excised specimen [6], when perhaps the assessment of unresected LNs may be more meaningful. Indeed, recent interest in CVL as a counterpart to CME suggests that an alternative approach to appraising the EoL would be useful [7, 8].

Based on the level of vascular ligation from the origin of the arterial vessel(s) supplying a particular segment of colon, CVL contributes to the completeness of a cancer operation [5]. This demarcates the maximum EoL by completely clearing regional LNs and those located centrally (to minimise any residual LN tissue potentially involved with cancer) [5, 9]. In cases where CVL is not strictly practised, pedicle ligation as high as possible is the usual aim. In both practices, the quality of lymphadenectomy may more accurately be determined by assessment of unresected residual LN tissue rather than the number of LNs found in the resected specimen.

The determination of whether CVL surgery has been performed remains largely dependent on corroboration by the surgical team. This practice does not allow for the level of pedicle transection to be assessed based upon pathological assessment of the excised specimen. Alternatively, in vivo RAPL measured on post-operative imaging has been proposed a more robust method to verify CVL surgery [7, 10, 11]. However, its role as a surrogate of the EoL and as a quality measure of survival outcomes in non-routine CVL practice has not been studied.

Therefore, the primary aim of this study was to assess the feasibility and reproducibility of measuring RAPL using surveillance CT in patients who had an AR or RH operation for CRC for which CVL was not routinely practised. A secondary aim of the study was to investigate whether RAPL was associated with survival outcomes or standard clinicopathological variables. We posited two hypotheses: (1) that measuring RAPL would prove to be both feasible and reliably reproducible and (2) a longer RAPL was associated with poorer oncological outcome and less radical lymphadenectomy.

## Materials and methods

**Fig. 1:**
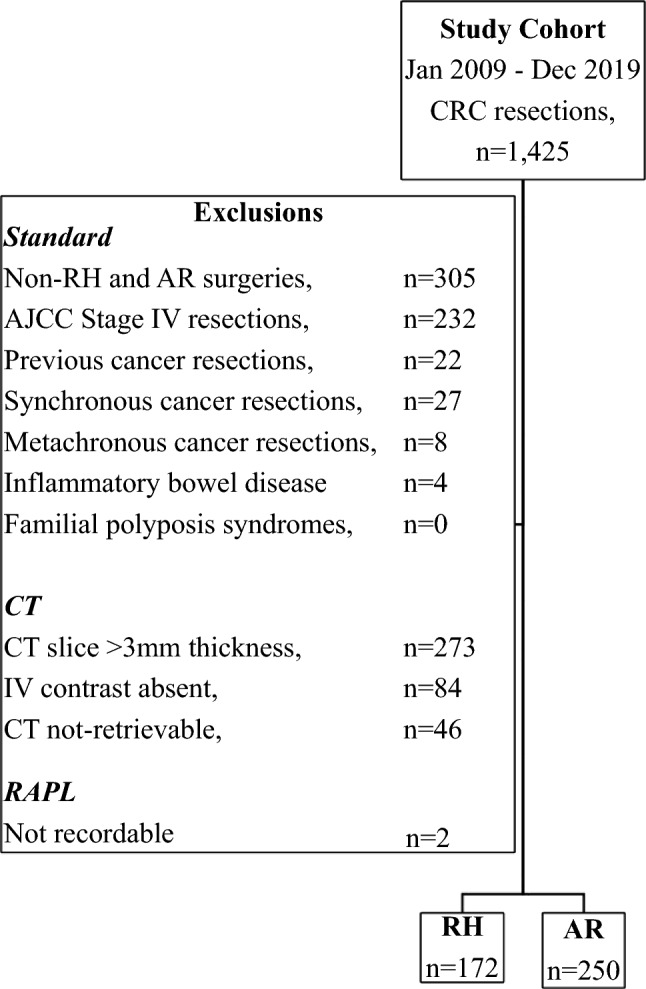
Flow diagram of cohort definition

A prospective observational cohort study of consecutive patients who underwent a potentially curative resection for a rectal or colon adenocarcinoma was performed. Patients included were those who had either an AR or RH performed between January 2009 and December 2019 at Concord Hospital, Sydney, Australia. Patients were identified from a prospectively maintained institutional database. Excluded were patients with American Joint Committee on Cancer (AJCC) Stage IV cancer, inflammatory bowel disease, polyposis coli, and a synchronous or metachronous CRC (Fig. 1).

### Surgical procedures

An AR operation was performed with adherence to TME principles and ligation of the IMA proximal to the origin of the ascending left colic artery (LCA), without abdominal aorta exposure [12]. A RH operation was performed according to the technique described by Bokey et al. [12], which involved ligation of the ICA without routine exposure of the superior mesenteric artery (SMA) or vein (SMV). In our unit, some surgeons perform a full lymphadenectomy but preserve the vascular stump by sweeping potential central nodes from the pedicle root distally in patients with suspected central lymphadenectomy. The precise tumour location was marked by the surgeon on a diagram including whether there was distant metastasis or unresected tumour remaining at the completion of the operation.

### Standard clinicopathological variables

Clinical information, operative details, tumour pathology, and follow-up data were obtained from the database for analysis [13, 14]. Specifically, details concerning the method of pathology reporting and staging have been previously described [15, 16].

### Clinico-pathological variable of interest—CT measurement of IMA and ICA RAPL (Fig. 2)

**Fig. 2:**
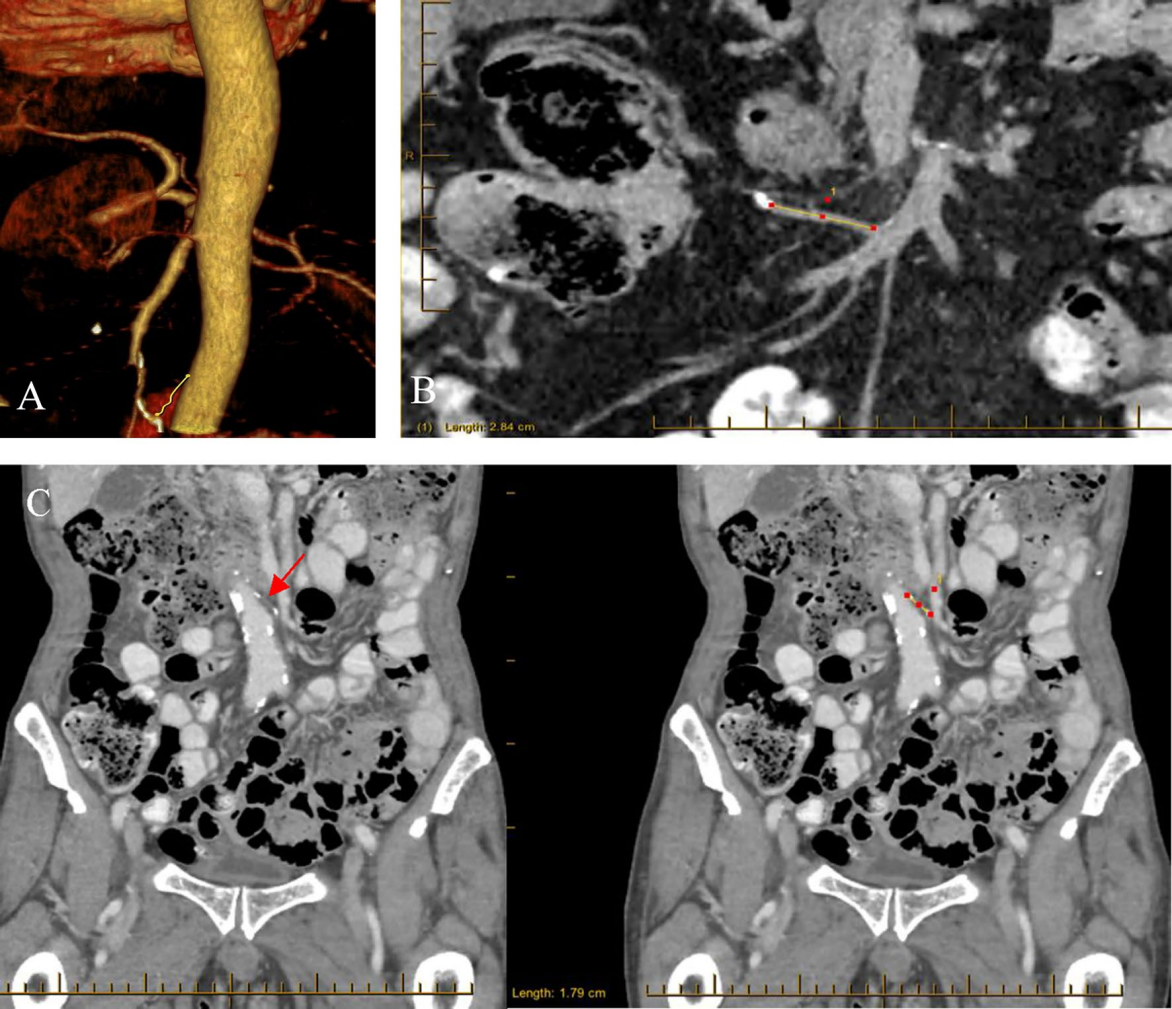
**A** Coronal volume-rendered reformatted CT image of the IMA RAPL, defined by its termination at a surgical clip; **B** Multi-planar reconstructed (MPR) CT image with ICA RAPL defined and measured in the coronal plane; **C** MPR CT image with IMA RAPL defined (red arrow) and measured in the coronal plane. Surgical clip/ in (**B**) and granuloma in (**C**) are denoted by hyper-density at maximal limits of the measurement

The RAPL of the post-AR IMA or post-RH ICA were prospectively measured and recorded in patients identified from the prospective database. The RAPL was positively identified if the location of a visible named vessel correlated to its expected anatomical position, or terminated at a surgical staple, surgical clip, or “radiological granuloma”. Patients whose RAPLs measured ≤ 10 mm were regarded as having had a CVL [5].

The IMA and ICA were chosen as the pedicles to measure as they correlated to the main feeding arteries in our two most commonly performed operations. On the left side, the IMA reliably originates from the abdominal aorta and assumes a subtle leftward course, following its origin at the level of the third lumbar vertebral body [10, 17]. Contralaterally, the ICA is the most constant collateral of the SMA, emerging usually approximately at the level of the fifth or sixth jejunal artery and assumes an antero-inferior trajectory [10].

Surveillance CT imaging up to 3 years post-surgery was used to standardize the timeline for RAPL measurement. Helical imaging acquisition of the abdomen and pelvis obtained during either the venous or arterial (if available) phase was used for multiplanar reconstruction or volume-rendered reformat of the mesenteric vasculature, using the syngo^®^.via (Siemens Healthcare, Germany) platform. The CTs were reviewed by KN, and once best displayed, a calliper tool was used to measure the post-resection arterial stump lengths. Patients were excluded from the study if their CT scans had missing or inconclusive results, specifically when the RAPL could not be easily traced because of slice thickness or the absence of intravenous contrast, as shown in Fig. 1. The RAPL measurements were repeated randomly in 10% of the cases by a (blinded) specialist gastrointestinal radiologist (JY) to determine the reproducibility of the measurements and overall inter-observer reliability.

### Pathology reporting and staging

Specialist pathologists examined resected specimens using a standard synoptic protocol, with pathology data coded by CC. Adenocarcinomas including mucinous and signet ring types were analysed. Fat clearance techniques were not employed in node retrieval. Tumour staging followed the AJCC pTNM system.

### Surveillance and follow-up

Patients were reviewed at least 6 monthly for the first 2 years after resection and followed up yearly thereafter until death or December 2021, unless lost to follow-up [18]. The approach to surveillance and indications for post-operative adjuvant chemotherapy have been previously detailed [16].

The date of resection was the start of the follow-up period. Follow-up times were censored at the last contact for patients who did not experience the terminal event up to December 2021, were lost to follow-up, or remained alive. The approach to identifying the date and cause of death have been described previously [16, 19].

### Outcome measures

The primary outcome measure focused on assessing the reproducibility of CT-based RAPL measurements in terms of reliability and agreement. Secondary outcome measures included OS, DFS, and any locoregional recurrence (LR) or systemic recurrence (SR) within the peritoneal cavity or elsewhere, and biopsy proven whenever possible [20–22].

### Statistical analyses

Continuous variables were reported as mean (standard deviation [SD]) for normally distributed variables and as median (interquartile range [IQR] or range [minimum to maximum values]) for non-normal distributions. Categorical variables were reported as frequencies and percentages. The inter-observer reliability of RAPL measurements was tested using intra-class correlation coefficient (ICC) modelling. Univariate and multivariate linear regression models tested correlation between RAPLs and clinicopathological factors. Survival and recurrence estimates were modelled using the Kaplan-Meier function with log-rank test performed to determine difference in survival distributions. Cox regression modelling tested for associations between outcome measures and relevant clinicopathological variables, including RAPL measurements. A multivariate model was not performed because multiple variables violated the proportional hazards assumptions, and alternative stratification of these violating variables rendered too small a case load for analysis. The level for two-tailed statistical significance was *P* < 0.05 with confidence intervals at the 95% level. All the analyses were performed using SPSS^®^ version 29 (IBM, New York, USA).

#### Sample size

Sample size calculations were made based on a previously reported 5-year DFS of 59.4% [16].

A relative improvement of 10% (attributable to CVL) in the 5-year DFS was deemed clinically relevant. Estimating that 5% of our patients had a CVL resection in our institution, and calculating for a two-sided significance of 0.05 (*alpha* of 0.05 and power of 0.85), a minimum of 415 patients were required for adequate power [23].

#### AJCC stage III sub-group analysis

It was expected that the greatest survival advantage for patients undergoing CVL surgery would be observed in the AJCC stage III population. To understand the survival association between RAPL and Stage III disease, a sub-group analysis was performed on this population, alongside a separate Cox regression survival analysis.

## Results

### Study population

A total of 1425 patients had a CRC resection during the study period. Of these, 827 were suitable for analysis. We reviewed post-operative CTs for 424 consecutive patients (Fig. 1) of which 422 (99.5%) had a recordable RAPL. Of these 422 patients, 250 (59.2%) and 172 (40.8%) patients had AR and RH surgery, respectively. Detailed clinico-pathological characteristics of AR and RH patients are shown in Table 1.

**Table 1:** Comparison of clinicopathological factors between patients with AR and RH

Variables	Total (%) or mean (SD) or median (range/IQR) (*n* = 422)	Anterior resection (*n* = 250)	Right hemicolectomy (*n* = 172)
RAPL (median [IQR]), mm*	27.2 (19.8–38.1)	26.4 (19.2–34.3)	29.8 (21.6–40.9)
CVL (RAPL < 10 mm)			
No	407 (96.4)	239 (95.6)	168 (97.7)
Yes	15 (3.6)	11 (4.4)	4 (2.3)
Gender			
Male	228 (54.0)	144 (57.6)	84 (48.8)
Female	194 (46.0)	106 (42.4)	88 (51.2)
Age (mean [SD]), years	69.0 (12.3)	66.3 (12.1)	72.8 (11.4)
BMI (mean [SD]), kg/m^2^	27.5 (5.8)	27.3 (5.9)	27.9 (5.6)
ASA grade			
I	61 (14.4)	44 (17.6)	17 (9.9)
II	229 (54.3)	138 (55.2)	91 (52.9)
III/IV	132 (31.3)	68 (27.2)	64 (37.2)
Duration of operation (median [IQR]), min	221.0 (171.5–274.5)	252.0 (215.0–302.0)	169.0 (143.75–201.0)
Emergency operation			
No	396 (93.8)	235 (94.0)	161 (93.6)
Yes	26 (6.2)	15 (6.0)	11 (6.4)
Operation modality			
Open	81 (19.2)	57 (22.8)	24 (14.0)
Laparoscopy	341 (80.8)	193 (77.2)	148 (86.0)
Procedure conversion			
No	312 (91.5)	173 (89.6)	139 (93.9)
Yes	29 (8.5)	20 (10.4)	9 (6.1)
Blood loss (ml)			
≤ 500	408 (96.7)	239 (95.6)	169 (98.3)
> 500	14 (3.3)	11 (4.4)	3 (1.7)
Tumour stage (TNM AJCC)			
Node negative (stage 1 and 2)	184 (43.6)	104 (41.6)	80 (46.5)
Node positive (stage 3)	238 (56.4)	146 (58.4)	92 (53.5)
Tumour size (median [IQR]), mm	4.0 (3.0–5.0)	3.8 (2.9–5.0)	4.5 (3.1–6.4)
Tumour perforation			
No	417 (98.8)	247 (98.8)	170 (98.8)
Yes	5 (1.2)	3 (1.2)	2 (1.2)
Histological type			
Non-mucinous or signet ring	381 (90.3)	237 (94.8)	144 (83.7)
Mucinous or signet ring	41 (9.7)	13 (5.2)	28 (16.3)
Histological differentiation			
Well or moderate	365 (86.5)	235 (94.0)	130 (75.6)
Poor	57 (13.5)	15 (6.0)	42 (24.4)
Histological grade			
Low or average	361 (85.5)	234 (93.6)	127 (73.8)
High	61 (14.5)	16 (6.4)	45 (26.2)
Lympho-vascular invasion			
No	291 (69.0)	173 (69.2)	118 (68.6)
Yes	131 (31.0)	77 (30.8)	54 (31.4)
Peri-neural invasion			
No	310 (73.5)	170 (68.0)	140 (81.4)
Yes	112 (26.5)	80 (32.0)	32 (18.6)
Number of lymph nodes examined (median [range])	19 (4–65)	19 (4–65)	20 (6–50)
Lymph node harvest (< 12)			
No	388 (91.9)	225 (90.0)	163 (94.8)
Yes	34 (8.1)	25 (10)	9 (5.2)
Time to surveillance CT from index operation (median [IQR), months	11.3 (7.3–16.7)	11.4 (7.3–17.7)	11.0 (7.1–14.7)
LOS (median [range]), days	7 (2–77)	7 (2–66)	6 (3–77)

*RAPL* residual arterial pedicle length, *CVL* central vascular ligation, *ASA* American Society of Anesthesiology, *TNM* 8th edition tumour, nodes, and metastasis staging system, *AJCC* American Joint Committee on Cancer, *LOS* length of stay

*A longer RAPL was noted in those who underwent RH surgery (*P* = 0.02)

**Fig. 3:**
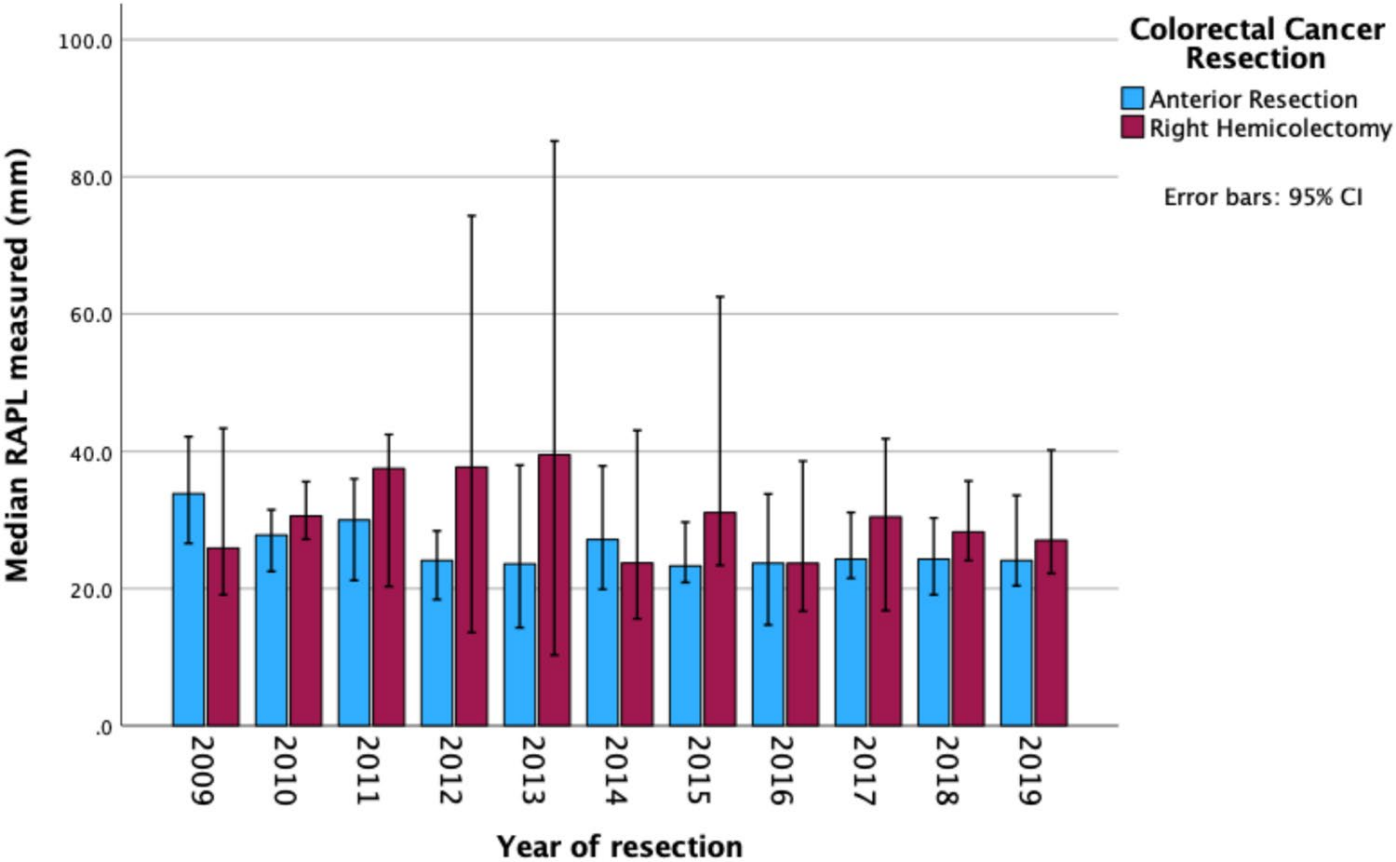
Trend of RAPLs over the 11-year study period in patients undergoing an AR or RH for colorectal cancer

The temporal trend in RAPL measurements is presented in Fig. 3. The median RAPL of patients who underwent an AR or RH was 26.4 (IQR 19.2–34.3) mm and 29.8 (IQR 21.6–40.9) mm, respectively. Eleven AR (4.4%) and four RH patients (2.3%) had CVL (i.e., RAPL ≤ 10 mm) performed.

The absolute inter-observer agreement for AR and RH RAPLs was 94.6% and 78.9%, respectively. Inter-rater reliability assessed by ICC was 0.97 (95% CI 0.93–0.99; *P* < 0.001) and 0.89 (95% CI 0.64–0.96; *P* < 0.001) in AR and RH patients, respectively.

### Comparison of survival outcomes between clinicopathological characteristics in AR and RH patients (Table 2)

**Table 2:** Comparison of oncological outcomes between clinicopathological features of AR and RH patients

	Overall survival				Disease-free survival			
	Anterior resection		Right hemicolectomy		Anterior resection		Right hemicolectomy	
	HR (95% CI)	*P*-value	HR (95% CI)	*P*-value	HR (95% CI)	*P*-value	HR (95% CI)	*P*-value
RAPL (mm)	1.01 (0.99–1.02)	0.14	1.01 (0.99–1.03)	0.15	1.01 (0.99–1.02)	0.26	1.01 (0.99–1.03)	0.12
CVL								
No	Ref	–	Ref	–	Ref	–	Ref	–
Yes	1.70 (0.62–4.67)	0.31	1.96 (0.48–8.04)	0.35	1.87 (0.76–4.61)	0.18	1.56 (0.38–6.38)	0.54
Gender								
Male	Ref	–	Ref	–	Ref	–	Ref	–
Female	0.57 (0.34–0.95)	0.03	0.66 (0.39–1.12)	0.12	0.63 (0.41–0.99)	0.045	0.77 (0.48–1.24)	0.77
Age (years)	1.05 (1.03–1.08)	< 0.001	1.05 (1.02–1.08)	0.001	1.03 (1.01–1.05)	0.003	1.03 (1.01–1.06)	0.01
BMI (kg/m^2^)	1.01 (0.97–1.05)	0.78	0.99 (0.94–1.04)	0.61	1.01 (0.98–1.04)	0.55	0.99 (0.95–1.04)	0.81
ASA grade								
I	Ref	–	Ref	–	Ref	–	Ref	–
II	2.15 (0.90–5.15)	0.09	0.99 (0.34–2.90)	0.98	1.99 (0.94–4.25)	0.07	1.43 (0.54–4.35)	0.43
III/IV	6.29 (2.59–15.28)	< 0.001	3.13 (1.11–8.86)	0.03	4.94 (2.27–10.73)	< 0.001	3.14 (1.11–8.86)	0.03
Emergency operation								
No	Ref	–	Ref	–	Ref	–	Ref	–
Yes	1.31 (0.47–3.60)	0.61	1.42 (0.51–3.92)	0.50	1.06 (0.43–2.62)	0.90	1.43 (0.58–3.57)	0.44
Operation modality								
Open	Ref	–	Ref	–	Ref	–	Ref	–
Laparoscopy	0.61 (0.37–1.01)	0.05	0.39 (0.22–0.71)	0.002	0.66 (0.42–1.03)	0.07	0.27 (0.16–0.46)	< 0.001
Intraoperative blood loss (ml)								
≤ 500	Ref	–	Ref	–	Ref	–	Ref	–
> 500	1.56 (0.62–3.91)	0.34	9.77 (2.23–42.85)	0.003	1.34 (0.54–3.32)	0.53	4.19 (1.00–18.48)	0.049
Tumour stage (TNM)								
Stage 1/2	Ref	–	Ref	–	Ref	–	Ref	–
Stage 3	2.08 (1.21–3.58)	0.008	1.57 (0.91–2.68)	0.10	1.80 (1.14–2.85)	0.01	1.89 (1.15–3.10)	0.01
Tumour size (cm)	1.13 (0.98–1.30)	0.09	1.08 (1.01–1.15)	0.02	1.16 (1.03–1.30)	0.02	1.11 (1.06–1.17)	< 0.001
Histological type								
Non-mucinous/signet ring	Ref	–	Ref	–	Ref	–	Ref	–
Mucinous/signet ring	1.21 (0.44–3.33)	0.71	1.91 (1.02–3.56)	0.04	2.04 (0.94–4.42)	0.07	1.83 (1.03–3.25)	0.04
Histological differentiation								
Well or moderate	Ref	–	Ref	–	Ref	–	Ref	–
Poor	2.31 (1.05–5.05)	0.04	1.21 (0.66–2.22)	0.54	2.13 (1.03–4.41)	0.04	1.37 (0.80–2.35)	0.26
Histological grade								
Low or average	Ref	–	Ref	–	Ref	–	Ref	–
High	3.00 (1.43–6.30)	0.004	1.18 (0.65–2.13)	0.58	3.07 (1.58–5.95)	< 0.001	1.32 (0.77–2.24)	0.31
Lympho-vascular invasion								
No	Ref	–	Ref	–	Ref	–	Ref	–
Yes	1.70 (1.04–2.78)	0.03	2.18 (1.27–3.74)	0.005	1.69 (1.10–2.60)	0.02	2.91 (1.78–4.75)	< 0.001
Peri-neural invasion								
No	Ref	–	Ref	–	Ref	–	Ref	–
Yes	2.21 (1.36–3.58)	0.001	1.18 (0.63–2.24)	0.61	2.51 (1.63–3.84)	< 0.001	1.59 (0.91–2.80)	0.10
Number of lymph nodes examined	0.98 (0.95–1.01)	0.15	0.97 (0.94–1.00)	0.08	0.99 (0.97–1.02)	0.57	0.98 (0.95–1.01)	0.20

*HR* hazard ratio, *RAPL* residual arterial pedicle length, *CVL* central vascular ligation, *ASA* American Society of Anesthesiology, *TNM* 8th edition tumour, nodes, and metastasis staging system, *AJCC* American Joint Committee on Cancer

**Fig. 4:**
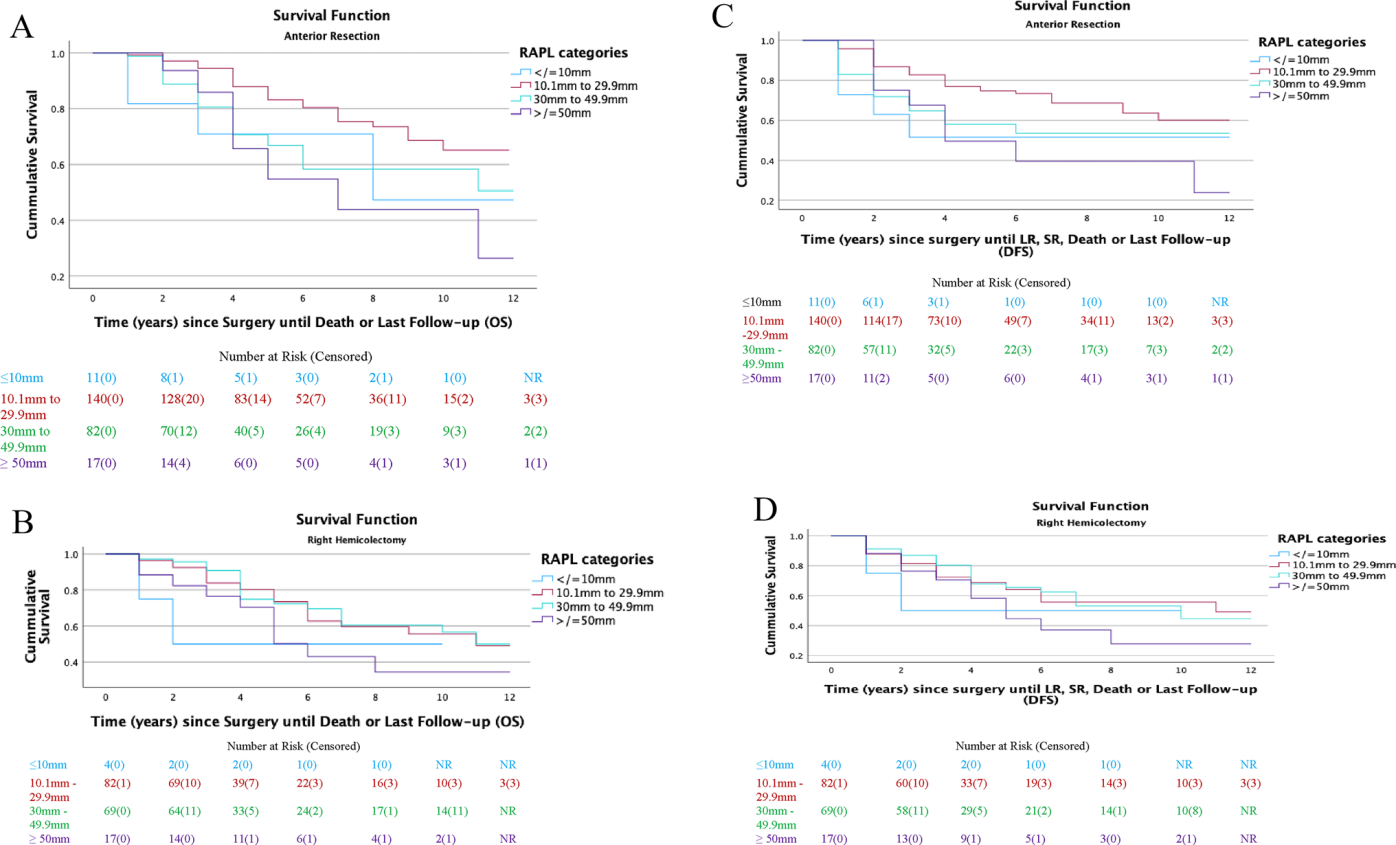
Kaplan-Meier plots of OS and DFS of AR and RH patients stratified by RAPLs. **A** OS of AR patients; **B** DFS of AR patients; **C** OS of RH patients; **D** DFS of RH patients

Table 2 summarizes the associations between clinicopathological characteristics and survival outcomes in patients who underwent AR and RH resections. Figure 4 presents the Kaplan-Meier plot of OS and DFS for these patients stratified according to RAPL.

#### Anterior resection

For those who underwent an AR, death occurred in 66 patients (26.4%). The 5-year OS and DFS rates were 75.6% (95% CI 72.5–78.7) and 66.6% (95% CI 63.4–69.8), respectively. A LR was diagnosed in 11 patients (4.4%), and SR was diagnosed in 58 patients (23.2%). The mean time to LR and SR was 1.5 (95% CI 1.0–1.9) years and 1.9 (95% CI 1.5–2.6) years, respectively.

RAPL was not associated with OS (*P* = 0.14) or DFS (*P* = 0.26), nor was CVL (OS [*P* = 0.31]; DFS [*P* = 0.18]). A poorer OS was associated with increasing age (*P* < 0.001), ASA > 2 (*P* < 0.001), AJCC stage III tumours (*P* = 0.008), poorly differentiated (*P* = 0.04) and high grade (*P* = 0.004) tumours, the presence of lympho-vascular invasion (LVI [*P* = 0.03]), and perineural invasion (*P* = 0.001). Poor prognosis characterised by increased DFS hazards mirrored all of the above factors in addition to large tumours (*P* = 0.02).

#### Right hemicolectomy

Amongst the RH patients studied, death occurred in 57 patients (33.1%). The median DFS was 9.3 (95% CI 5.2–13.4) years. The 5-year OS and DFS rates were 69.6% (95% CI 65.5–73.7) and 61.8% (95% CI 57.6–66.0), respectively. A LR was diagnosed in three patients (1.7%) and SR diagnosed in 36 (20.9%). The median time to LR and SR was 2.8 years (95% CI 0.3–5.2) and 1.2 years (95% CI 0.7–1.6), respectively.

RAPL was not associated with OS (*P* = 0.15) or DFS (*P* = 0.12). Similarly, CVL was also not associated with OS (*P* = 0.35) or DFS (*P* = 0.54). Poorer OS was associated with increasing age (*P* = 0.001), ASA score > 2 (*P* = 0.03), intraoperative blood loss > 500 ml (*P* = 0.003), larger tumours (*P* = 0.02), mucinous or signet cancers (*P* = 0.04), and LVI (*P* = 0.005). Regarding DFS, a poorer prognosis mirrored all factors noted above.

### Clinico-pathological characteristics and RAPL associations in AR and RH patients (Table 3A, B)

**Table 3:** Clinicopathological characteristics and RAPL associations in AR and RH patients  (3A—univariate; 3B—multivariate analyses)

3A				
	Residual arterial pedicle length (mm)			
	Anterior resection (*n* = 250)		Right hemicolectomy (*n* = 172)	
	*β* (95% CI)	*P*-value	*β* (95% CI)	*P*-value
Year of operation (year)	− 0.59 (− 1.13 to − 0.06)	0.03	− 0.54 (− 1.20 to 0.13)	0.11
Gender	− 4.71 (− 8.46 to − 0.96)	0.01	− 6.87 (− 11.29 to − 2.45)	0.002
Male				
Female				
Age (years)	0.06 (− 0.09 to 0.22)	0.43	0.27 (0.07 to 0.47)	0.008
BMI (kg/m^2^)	0.34 (0.14 to 0.66)	0.04	0.30 (− 0.12 to 0.71)	0.16
ASA grade	3.66 (0.87 to 6.46)	0.01	5.26 (1.75 to 8.78)	0.004
I				
II				
III/IV				
Emergency operation	3.43 (− 4.46 to 11.32)	0.39	3.90 (− 5.36 to 13.16)	0.41
No				
Yes				
Operation modality	− 9.45 (− 13.77 to − 5.14)	< 0.001	− 7.75 (− 14.20 to − 1.31)	0.02
Open				
Laparoscopy				
Intraoperative blood loss (ml)	3.43 (− 5.71 to 12.57)	0.46	10.95 (− 6.31 to 28.21)	0.21
≤ 500				
> 500				
Tumour stage (TNM AJCC)	− 0.06 (− 3.86 to 3.75)	0.98	− 0.26 (− 4.81 to 4.29)	0.91
Stage 1/2				
Stage 3				
Tumour size (cm)	0.29 (− 0.72 to 1.31)	0.57	0.04 (− 0.65 to 0.73)	0.91
Histological type	2.40 (− 6.08 to 10.81)	0.58	6.05 (− 0.03 to 12.13)	0.05
Mucinous or signet ring				
Non-mucinous or non-signet ring				
Histological differentiation	− 3.02 (− 10.92 to 4.87)	0.45	0.35 (− 4.93 to 5.63)	0.90
Well or moderate				
Poor				
Histological grade	− 0.61 (− 8.27 to 7.06)	0.88	1.88 (− 3.28 to 7.04)	0.47
High				
Low or average				
Lympho-vascular invasion	− 0.55 (− 4.62 to 3.51)	0.79	− 2.21 (− 7.09 to 2.67)	0.37
No				
Yes				
Peri-neural invasion	0.66 (− 3.37 to 4.68)	0.75	− 1.17 (− 7.00 to 4.66)	0.69
No				
Yes				
Number of lymph nodes examined	− 0.07 (− 0.28 to 0.15)	0.54	− 0.20 (− 0.48 to 0.08)	0.16
Time to surveillance CT from index operation	0.05 (− 0.16 to 0.25)	0.66	− 0.18 (− 0.46 to 0.09)	0.19
LOS (days)	0.11 (− 0.14 to 0.35)	0.38	0.07 (− 0.16 to 0.30)	0.56

3B				
	Residual arterial pedicle length (mm)			
	Anterior resection (*n* = 250)		Right hemicolectomy (*n* = 172)	
	*aβ* (95% CI)	*P*-value	*aβ* (95% CI)	*P*-value
Year of operation (year)	− 0.23 (− 0.82 to 0.35)	0.44		
Gender	− 3.49 (− 7.23 to 0.24)	0.07	− *5.67 (*− *10.05 to *− *1.29)*	*0.01*
Male				
Female				
Age (years)			0.16 (− 0.05 to 0.37)	0.14
BMI (kg/m^2^)	0.22 (− 0.10 to 0.54)	0.17		
ASA grade	*3.08 (0.14 to 6.03)*	*0.04*	2.94 (− 0.89 to 6.77)	0.13
I				
II				
III/IV				
Operation modality	− *8.70 (*− *13.51 to *− *3.89)*	< *0.001*	− 4.91 (− 11.30 to 1.48)	0.13
Open				
Laparoscopy				

*RAPL* residual arterial pedicle length, *CVL* central vascular ligation, *ASA* American Society of Anesthesiology, *TNM* 8th edition tumour, nodes, and metastasis staging system, *AJCC* American Joint Committee on Cancer, *CT* computed tomography, *LOS* length of stay

#### Anterior resection

On univariate analysis, a shorter RAPL was associated with female patients (*P* = 0.01), laparoscopic operation (*P* < 0.001), and more recent study years (*P* = 0.03). A longer RAPL was associated with a higher BMI (*P* = 0.04) and ASA grades (*P* = 0.01). RAPL was not associated with the number of LNs examined (*P* = 0.54). On multivariate analysis, only ASA grade (*P* = 0.04) was significantly associated with a longer RAPL and laparoscopic operation (*P* < 0.001) with a shorter RAPL.

#### Right hemicolectomy

RH patients had a longer RAPL than AR patients (Table 1). On univariate analysis, a shorter RAPL was observed in female patients (*P* = 0.002) and laparoscopic operation (*P* = 0.02). A longer RAPL was associated with increasing age (*P* = 0.008) and a high ASA grade (*P* = 0.004). Again, RAPL was not associated with the number of LNs examined (*P* = 0.16). On multivariate analysis, only gender (*P* = 0.01) was associated with a shorter RAPL.

### AJCC stage III sub-group analysis

There were 238 AJCC Stage III patients with a recorded RAPL. Of these, 146 had an AR and 92 had a RH. The median RAPLs of patients who underwent an AR and RH were 26.7 (IQR 19.5–36.3) mm and 29.8 (IQR 21.2–40.5) mm, respectively. Within this cohort, six AR patients (4.1%) and three RH patients (3.3%) had a CVL performed.

The survival associations of the sub-cohort are summarised in Table 4. In patients who underwent an AR, RAPL was associated with poorer OS (*P* = 0.04) but not DFS (*P* = 0.10). In those who had a RH, RAPL was associated with neither OS (*P* = 0.27) nor DFS (*P* = 0.64).

**Table 4:** Oncological associations between AJCC Stage 3 patients and RAPL (*N* = 238)

	Overall survival				Disease-free survival			
	Anterior resection		Right hemicolectomy		Anterior resection		Right hemicolectomy	
	HR (95% CI)	*P*	HR (95% CI)	*P*	HR (95% CI)	*P*	HR (95% CI)	*P*
RAPL (mm)	1.03 (1.00–1.05)	0.04	1.01 (0.99–1.03)	0.27	1.02 (0.99–1.04)	0.10	1.01 (0.99–1.02)	0.64

*HR* hazard ratio, *RAPL* residual arterial pedicle length

## Discussion

Over a 10-year period, this large and adequately powered prospective cohort study evaluated the role of RAPL measured on surveillance CT as a marker of surgical quality and EoL. This was achieved by investigating the association of RAPLs with survival outcomes and other clinicopathological variables in patients who had either an AR or RH for CRC. This study demonstrates the feasibility and reproducibility of measuring RAPLs in patients in whom CVL surgery was not routinely performed. Although some clinicopathological factors were individually associated with poorer survival outcomes, RAPLs showed no influence on these outcomes. Notably, RAPL was not associated with the extent of the LN harvest. These findings suggest that using RAPL as a quality marker for CRC when measured in a non-routine CVL population is of minimal value in predicting patient outcome.

The current assessment of the extent of lymphadenectomy in CRC surgery relies on rudimentary measures, such as LN harvest, and a nodal count of 12 is widely accepted as an adequate lymphadenectomy for staging purposes [24, 25]. Measurements based on tissue morphometry, such as the area of mesentery excised, have been previously described and proposed as objective measures for lymphadenectomy extent [9]. Nevertheless, when considering lymphadenectomy for the purpose of ensuring all potentially involved nodes are excised, of which CVL is a possible ‘gold standard’, there should be a focus on quantifying the unresected nodal tissue remaining. Neither LN harvest nor tissue morphometry measurements of the pathology specimen can provide this assessment. Our study investigated whether in vivo measurement of RAPLs using routine surveillance CT could fill this role by providing an objective assessment of residual nodal tissue using RAPL as a surrogate marker.

This study confirmed the feasibility and reproducibility of measuring IMA and ICA pedicle length following AR and RH, respectively. IMA and ICA pedicles were consistently identified in > 400 postoperative scans, with identification aided through recognition of a surgical clip/staple, or “radiological granuloma”. The reproducibility of these measurements was confirmed by a second independent observer demonstrating good inter-observer agreement. Notably, IMA pedicle lengths were, on average, shorter than ICA pedicle lengths, likely a reflection of the accepted and inherent complexity seen in the vascular anatomy of the right colon compared to the left. The difference in IMA and ICA pedicle lengths justifies our approach of considering AR and RH patients separately in a study designed to evaluate the association between pedicle length and survival.

The measurement of RAPL following CRC surgery has previously been described but with heterogenous study populations, study designs, and imaging protocols [10, 11, 26–28]. One previous study measured ICA stump length following RH surgery many months after the index operation [26] but did not investigate its relationship with survival outcomes. In another study, where radio-surgical correlation of IMA ligation was noted in only 41% of patients, no survival difference between the presence or absence of radio-surgical correlation of IMA ligation was observed [28]. In a separate registry-based study, Bostrom et al. highlighted no survival difference between the level of vascular tie when it was dichotomised to high and low ligation. These authors did not record individual RAPLs [29]. Only one study has previously investigated the relationship between RAPL and survival outcome, but in a cohort where CVL was routinely intended to be practiced. In that study, a pedicle length of < 1 cm (verifying CVL had been performed) was associated with survival benefit [7]. Our study aimed to determine whether a similar survival benefit was demonstrable in a cohort where CVL was not routinely practiced. Our original hypothesis that a shorter RAPL would confer some survival benefit was not substantiated, as there was no association between RAPL and OS or DFS in either AR or RH patients.

The result of our subgroup analysis warrants comment. When confined to Stage III patients alone, there was an apparent association between RAPL and OS, but not DFS, in AR patients. This association, however, was weak and is difficult to explain. It is challenging to resolve why a shorter RAPL would provide an OS benefit without improvement in DFS. Notably, this significant association was observed only on univariate analysis.

Our study was unable to demonstrate a clear association between RAPL and LN harvest. A more radical operation has been associated with a larger LN harvest in previous studies [8, 30, 31], so intuitively, an association between a shorter RAPL and a larger LN harvest should be expected. We suppose two reasons for this observation. The first relates to the surgical technique practiced within our unit, whereby some surgeons perform a full lymphadenectomy but preserve the vascular stump by sweeping central nodes from the pedicle root distally. This may result in a longer RAPL despite a complete lymphadenectomy being performed. Second, pursuing a higher vascular tie may not necessarily translate into acquiring a larger nodal harvest. In a previous study from our unit investigating the relationship between apical node positivity and survival outcome [32], over one-third of resected CRC specimens did not have an apical node present (defined as a node within 1 cm of the pedicle ligature), suggesting that extending pedicle transection proximally may not necessarily result in a greater (apical) LN yield.

Although this study was unable to demonstrate an association between RAPL and survival outcomes or LN yield, its clinical implications are important. Only one previous study used a methodology comparable to ours, but in a cohort of patients where CVL was routinely intended to be performed, and found a survival benefit with a shorter RAPL, specifically where CVL was verified [7]. The fact that our study was unable to replicate those results in a cohort of non-routine CVL implies the benefits of a shorter RAPL may only be reaped when a CVL is performed (RAPL ≤ 10 mm) and that probably CVL is an ‘all or nothing phenomenon’. Furthermore, the lack of association between RAPL and LN yield serves to emphasise the continuing importance of comprehensive pathology assessment of resected specimens, of which accurate nodal count remains highly relevant to cancer staging and guiding the need for adjuvant therapy.

This prospective study has several limitations. In patients who underwent a RH for a hepatic flexure malignancy, the ICA was regarded as the principal vascular pedicle to standardise the identification of a single pedicle radiologically, despite other pedicles sharing lymphatic drainage in this region. As not all patients had their heights recorded in the database, we were unable to adjust RAPL measurements according to the patients’ body mass indices. Additionally, in a unit where CVL was not routinely practiced, it is highly probable that aside from simply sweeping the central nodes up into the resection margin of the specimen without a high tie of the vessel, CVL was selectively performed in cases where central lymphadenopathy was suspected. This scenario presents a potential for introducing selection bias. However, the well-powered and large cohort size, long study duration, application of standardized surgery by specialist colorectal surgeons following anatomical planes, and routine detailed generic pathology reporting are strengths of our study. Additionally, the inclusion of urgent operations and minimally and maximally invasive surgical approaches improves the generalizability of our study.

## Conclusion

This study is the first to demonstrate the feasibility of measuring RAPL using surveillance CTs following non-routine CVL surgery for CRC in AR and RH patients. The null association between RAPL and survival outcomes questions the role of RAPL as a quality marker for CRC surgery in patients in whom CVL is not intended. The role of RAPL measurement may be confined to post hoc verification that CVL surgery has been performed, as this appears to be where survival benefits have been documented. Otherwise, accurate and structured pathology assessment of resected specimens remains crucial for disease prognostication and guidance of the need for adjuvant therapy in patients who have had a potentially curative operation for CRC.

## Data Availability

The data that support the findings of this study are available from the corresponding author, Dr Kheng-Seong Ng, upon reasonable request.
